# “In my age, we didn’t have the computers”: Using a complexity lens to understand uptake of diabetes eHealth innovations into primary care—A qualitative study

**DOI:** 10.1371/journal.pone.0254157

**Published:** 2021-07-07

**Authors:** Catherine H. Yu, Maggie McCann, Joanna Sale

**Affiliations:** 1 Division of Endocrinology & Metabolism, Department of Medicine, St. Michael’s Hospital, Toronto, Ontario, Canada; 2 Li Ka Shing Knowledge Institute, St. Michael’s Hospital, Toronto, Ontario, Canada; 3 Faculty of Medicine, University of Toronto, Toronto, Ontario, Canada; 4 Dalla Lana School of Public Health, University of Toronto, Toronto, Ontario, Canada; University of Birmingham, UNITED KINGDOM

## Abstract

**Background:**

Shared decision-making is a central component of person-centred care and can be facilitated with the use of patient decision aids (PtDA). Barriers and facilitators to shared decision-making and PtDA use have been identified, yet integration of PtDAs into clinical care is limited. We sought to understand why, using the concepts of complexity science.

**Methods:**

We conducted 60-minute in-depth interviews with patients with diabetes, primary care physicians, nurses and dietitians who had participated in a randomized controlled trial examining the impact of *MyDiabetesPlan* (an online goal-setting PtDA). Relying on a qualitative description approach, we used a semi-structured interview guide to explore participants’ experiences with using *MyDiabetesPlan* and how it was integrated into the clinical encounter and clinical care. Audiotapes were transcribed verbatim, then coded independently by two analysts.

**Findings:**

17 interviews were conducted (5 physicians, 3 nurses, 2 dietitians, 7 patients). Two themes were developed: (1) *MyDiabetesPlan* appeared to empower patients by providing tailored patient-important information which engaged them in decision-making and self-care. Patients’ use of *MyDiabetesPlan* was however impacted by their competing medical conditions, other life priorities and socioeconomic context. (2) *MyDiabetesPlan* emphasized to clinicians a patient-centred approach that helped patients assume greater ownership for their care. Clinicians’ use of *MyDiabetesPlan* was impacted by pre-existing clinical tools/workplans, workflow, technical issues, clinic administrative logistics and support, and time. How clinicians adapted to these barriers influenced the degree to which *MyDiabetesPlan* was integrated into care.

**Conclusions:**

A complexity lens (that considers relationships between multiple components of a complex system) may yield additional insights to optimize integration of PtDA into clinical care. A complexity lens recognizes that shared decision-making does not occur in the vacuum of a clinical dyad (patient and clinician), and will enable us to develop a family of interventions that address the whole process, rather than individual components.

**Trial registration:**

ClinicalTrials.gov NCT02379078.

## Introduction

Diabetes is a prevalent chronic disease associated with complications such as cardiovascular disease which can be prevented or delayed with rigorous management of metabolic risk factors such as blood glucose, blood pressure and lipids [1]. Many strategies, both non-pharmacologic and pharmacologic, exist to target these risk factors [1, 2]. As such, patients are required to make multiple decisions throughout their life, yet are often ill-equipped to do so, due to limited knowledge, empowerment or opportunity [3]. Goal-setting is an integral component of person-centred care and effective diabetes care management, and has been demonstrated to improve A1c, patient provider relationships, and patient’s competence to manage diabetes [4]. Similarly, shared decision-making is a central component of person-centred care [5] and can be facilitated with the use of patient decision aids (PtDA) [6]. PtDA can increase patient knowledge, improve patient-clinician communication, and in diabetes care, increase those choosing to start new medications [6].

**Despite this, successful integration of PtDAs into clinical care continues to be elusive, with rates as low as 9.3% [7]. For example,** in our previous work, we conducted a cluster randomized controlled trial of *MyDiabetesPlan*, an web-based PtDA to facilitate goal-setting in patients with diabetes [8]. While single-decision PtDAs exist in diabetes care (e.g. whether to take a statin or not) [9], to our knowledge, a diabetes goal-setting PtDA that encompasses the patient’s life values and preferences as well as the multiplicity of comorbidities and management strategies does not exist. Given the complexity of decision-making in comprehensive diabetes care and the requirement for interactive user input, a web-based platform was chosen [10]. One of the objectives of this trial was to assess the feasibility of integrating *MyDiabetesPlan* into primary care, using trial logs and website usage logs to assess intervention fidelity and resource usage from our cluster randomized control trial. We recorded the clinical encounters in the intervention groups to identify barriers and facilitators to integration of *MyDiabetesPlan* into clinical care across the interprofessional team. A total of 179 appointments occurred (for 102 patients). Mean time for completion of *MyDiabetesPlan* by the clinician and the patient during initial appointment was 37 minutes. Barriers to use included those related to *MyDiabetesPlan* (e.g. electronic format), clinician (e.g. time required to complete), and patient factors (e.g. computer literacy). We concluded that *MyDiabetesPlan* use in clinical care was feasible. However, lower than expected numbers of diabetes-specific appointments were observed, and only 40 (36%) of patients completed *MyDiabetesPlan* at least twice. This suboptimal use occurred despite the research team undertaking an iterative user-centred design and evidence-based approach within a Knowledge-to-Action framework [11]. Throughout the design process, we engaged users (patients, clinicians), and conducted literature searches to identify barriers and facilitators to use, then refined *MyDiabetesPlan* based on this input [8, 10]. However, this approach was arguably “linear” and unidirectional, and did not fully consider the full context of the clinical encounter and how *MyDiabetesPlan’s* fit into the health care system and patients’ lives, nor the multi-directional interactions and relationship between these factors. As such, while instructive, it did not represent the whole picture of how to integrate a PtDA into clinical practice.

In contrast, the application of complexity science (which considers dynamic interactions between multiple components of a complex system) to understand this process may yield additional insights to optimize integration of PtDA into clinical care [12]. Health care is increasingly being recognized as complex. Acknowledgement of complexity will allow us to not only identify all system actors and system contexts for intervention use, but also understand the multiple relationships between them, and system behavior [13]. In doing so, complexity thinking supports patient-centered and intersectional approaches to care, and can inform novel out-of-box strategies for implementation and evaluation [14]. Furthermore, complexity science posits that despite the unpredictable nature of a complex system, actors within the system have the ability to “make sense” of the context, and act or “self-organize” in order to address, tackle or embrace the challenge or innovation [15]. Thus, adopting a complexity science lens to our enquiry would enable identification of the multidirectional relationships between *MyDiabetesPlan*, the patient, the clinician, the care team, the clinic, the health system and the patient’s life, and may highlight common themes that can be leveraged to enable successful scale-up.

The objective of this study was to explore the integration of *MyDiabetesPlan* into clinical care, through a complexity science lens, in order to identify opportunities for sense-making and self-organization that can be leveraged for scale-up and sustainability. To our knowledge, a qualitative exploration of the organizational barriers to implementation of a web-based interprofessional shared-decision-making PtDA in primary care has not previously been done and was thus undertaken.

## Methods

### Study overview

This study was part of a larger research program. Briefly, following the principles of user-centred design, we created *MyDiabetesPlan*, an online PtDA to facilitate goal-setting in patients with diabetes. Based on patient metabolic and behavioral data, values and preferences, *MyDiabetesPlan* provides a series of tailored strategies and management options for the patient, then synthesizes an action plan. Based on our feasibility and usability studies, we planned for initial completion of *MyDiabetesPlan* to occur with a member of the interprofessional health care team (e.g. nurse, dietitian) during the initial intake (e.g. support for entering in medications). We then tested its use in a 1-year cluster randomized controlled trial at 10 primary care sites in the Greater Toronto Area and surrounding areas and assessed its impact on decisional conflict.

#### Qualitative approach and research paradigm

We conducted a qualitative descriptive study with a naturalistic inquiry approach [16] using one-on-one interviews (face-to-face or telephone) with trial participants in the intervention sites after completion of the trial. Naturalistic inquiry aims to study individuals in their natural surroundings with minimal manipulation of the study setting [16]. We followed Standards for Reporting Qualitative Research [17]. We were informed by the concepts of complexity science [18] to identify key interdependencies and opportunities for self-organization and sense-making [15]. *Interdependencies* are defined as “relationships, connections and interactions among parts of a complex system, and are the structures and processes through which people interact.” [15]. From these patterns of interdependencies, we then identified patterns of self-organization (how people behave) as well as methods of sense-making (how people assign meaning to experience) [15]. Specifically, we framed our inquiry and analysis around identifying various actors involved in integrating *MyDiabetesPlan* into care as well as their interactions with each other. The topic areas in our interview guide soughtto understand how characteristics of various actors and their interactions with each other impacted use of *MyDiabetesPlan*. Finally, we tried to identify how actors “made sense” of *MyDiabetesPlan* integration and how their behaviors “self-organized” or promoted or impeded the use of *MyDiabetesPlan*.

### Setting and participants

Following completion of this trial, we invited all clinicians (n = 29) in the five intervention sites to participate in 60-minute individual in-depth interviews. We subsequently created a list of patients who had completed the trial (n = 102) and asked clinicians for recommendations on who to approach for interviews. Participants were contacted by their previously established preferred method of communication (email or telephone) and invited to participate. If interested, informed consent was obtained and an interview was scheduled in person (clinic, patient’s home) or by telephone.

### Data collection

Patients completed a sociodemographic questionnaire at trial initiation; clinicians completed a questionnaire regarding practice characteristics. Using similar interview guides for patients and clinicians (S1 and S2 Appendices), we examined how patients and clinicians approached decision-making and goal setting in general then if/how this was affected by *MyDiabetesPlan*. We elicited feedback regarding *MyDiabetesPlan* regarding its content, impact on addressing values and concerns, impact on flow of appointment, reasons for non-use as well as facilitators and barriers to sustained use. Finally, we inquired about the impact of an interprofessional approach and multimorbidity on *MyDiabetesPlan* use as potential facilitators and barriers. The interview guide was developed, reviewed and pilot tested on other members of the research team with expertise in diabetes care, interprofessional collaboration and shared decision-making and refined accordingly. Interviews were conducted by a team member (MM) with a medical background who was not involved in *MyDiabetesPlan* development nor the randomized controlled trial. We conducted interviews and analysis concurrently and refined the guide iteratively to explore new areas introduced by the interviewees. For example, earlier interviews identified that patient participants used *MyDiabetesPlan* as a conversation aid and behaviour change tool; the interview guide was amended to additionally explore these roles. Interviews were audiotaped then transcribed verbatim and deidentified.

### Data analysis

Transcripts were coded independently by two analysts. Any discrepancies in codes were reviewed and agreed on by consensus by the two coders; if the code could not be agreed upon, a third party reviewed the code and decided. Codes were created based on the content presented in the transcripts and refined in discussion with the research team, which included a team member with extensive expertise in qualitative methodology (JS). The coding template was then applied to the data and iteratively refined from ongoing interviews. Subsequently, themes were developed from these codes based on the concepts of complexity science. To ensure analytic trustworthiness, we created an audit trail to document decisions made during analysis [19]. As well, we practiced theoretical consistency by combining qualitative description with naturalistic inquiry, provided direct quotations to support the themes, employed the use of multiple analysts, and searched for and discussed negative cases during the development of themes [20].

### Research ethics

The study was approved by the Research Ethics Boards of Markham Stouffville Hospital (CIHR protocol, v2, January 2016), North York General Hospital (13–0265), St. Michael’s Hospital (13–014), University Health Network (16–6044), and Women’s College Hospital (2014-0043-B).

## Results

### Participant characteristics

A total of 7 patients and 10 clinicians (5 physicians, 3 nurses, 2 dietitians) were interviewed (S1 and S2 Tables). Of the 102 patients who completed the trial, clinicians identified 20 potential interviewees; of these, 7 agreed to participate. Reasons for non-participation included non-response, lack of time and unavailability due to competing medical illness. Of the 29 clinicians approached, 10 agreed to participate; reasons for non-participation included non-response and lack of time.

### Findings

We present our findings in 2 main themes (additional illustrative quotes in Table 1):

1. *MyDiabetesPlan* appeared to empower patients by providing tailored patient-important information which engaged them in decision-making and self-care. Patients’ use of *MyDiabetesPlan* was however impacted by their competing medical conditions, other life priorities and socioeconomic context.
2. *MyDiabetesPlan* emphasized to clinicians a patient-centred approach that helped patients assume greater ownership for their care. Clinicians’ use of *MyDiabetesPlan* was impacted by pre-existing clinical tools/workplans, workflow, technical issues, clinic administrative logistics and support, and time. How clinicians adapted to these barriers influenced the degree to which *MyDiabetesPlan* was integrated into care.

**Table 1:** Additional selected quotes illustrating themes.

Theme	Quote
**Theme 1.** MyDiabetesPlan appeared to empower patients by providing tailored patient-important information which engaged them in decision-making and self-care. Patients’ use of MyDiabetesPlan was however impacted by their competing medical conditions, other life priorities and socioeconomic context.	
MyDiabetesPlan allowed for individualized patient-centred goal-setting	“We are all different and we don’t all have to be size 10 or 11. I think that is a pretty realistic outlook” (C044, 74-year-old female patient)
MyDiabetesPlan engaged the patient in their care	“People probably would never think about what activities they like to do and how that may be impacted by a complication. It was a nice tie in of the two. To make it more meaningful for the patient.” (HCP071, RD in practice for 32 years)
Patients were empowered to take an active role in their decision-making	“It gave me more information so that when I talked to my doctor, I knew more about what I needed to do.” (K017, 50-year-old male patient)
MyDiabetesPlan stimulated patient engagement and fruitful discussion	“I think it’s a great way to have the discussion, and the discussion is the most important part, right? It’s such a neat way to have an appointment. The discussion is different from what we have when a patient just comes in and sits in the chair.” (HCPC050, RD in practice for 20 years)
MyDiabetesPlan enabled clinicans to incorporate new aspects to diabetes care	“I think that having people on their own articulate what their concerns are–what their big concerns are–are interesting. […] Our people with diabetes have high rates of depression, but I feel that sometimes you don’t always ask about mood and stuff like that. That tools you know [are] going to kind of bring out issues that potentially might just slip under the radar ‘cause you get a bit inured to [them].” (HCP025, FD in practice for 37 years)
	“The tool was really good because it offered lots of changes and it offered lots of options. It also added things that I may have not thought about.” (HCP037, RN in practice for 39 years)
**Theme 2.** Clinician use of *MyDiabetesPlan* was impacted by pre-existing clinical tools/workplans, workflow, technical issues, clinic administrative logistics and support, and time. How clinicians adapted to these barriers influenced the degree to which *MyDiabetesPlan* was integrated into care	
Use of *MyDiabetesPlan* was crowded out by pre-existing tools and workflow.	“I think that almost everybody that was in this [study], we’ve already gone through our own tool, so we have an idea of what we would be suggesting and what they’re doing.” (HCP071, RD in practice for 32 years)
Clinicians did not have their patients return because they ran out of time to complete *MyDiabetesPlan*	“I’m not gonna ask them to come back again to do a foot exam and a blood pressure and check their meters, so sometimes it would get added on at the end of the appointment or it just didn’t get done.” (HCP037, RN in practice for 39 years)
Technical issues relating to accessibility hampered its’ use by clinicians	“The biggest hurdle was just trying to access it. It was really hard to access. So I know there was a link embed in our EMR, but it often did not click through and open up the tool. I wasn’t gonna take appointment time to try and figure it out. If it wasn’t there right away, I didn’t press on.” (K074, FD in practice for 22 years)
Recalling patients for regularly scheduled diabetes-specific visits was administratively complex	“I don’t have diabetes visits–I have visits.” (HCP B023, FD in practice for 7 years)
	“I suppose when I saw them, but I haven’t been there for a number of months.” (K002, 74-year-old male patient)
	“The pharmacist was involved to some extent. The nurses were involved. And I know that administrative staff were involved in trying to make sure that the bookings were appropriate and getting in touch with patients. There was a lot of people involved in trying to make sure that this worked.” (HCP003, FD in practice for 9 years)
Providing diabetes care took time	“What’s your glucometer? Take your shoes and socks off and let me look at your feet. A lot of this was done over the winter and so you get six layers, and it takes time, right? To get the boots off, and the socks, and the long underwear. Like it just takes forever, right? So it was hard to integrate and so I think for those patients they just didn’t get that piece.” (HCP037, RN in practice for 39 years)
Particular moments in the patient’s disease and life trajectory may call for use of *MyDiabetesPlan*.	“So early on, people go “Yeah yeah Yeah, I wanna do that!” and then over time, they drop doing it. […] People who have had diabetes for a while, if they’re a bit frustrated and you give them a new tool that’s easy to use–that helps them to kickstart something again that might be helpful. In the case of the guy that I had with depression, I’m not sure about that, but [sigh] if I had been smarter or whatever, maybe that would’ve been actually a good thing for him to do because he would’ve engaged with it. […] I think there’s a timing for it that’s important.” (HCP025, FD in practice for 37 years)
Clinicians pondered over distributing *MyDiabetesPlan* over several appointments	“My only comment with *MyDiabetesPlan* is it’s long. […] I wonder if there’s a way for some of the portions here [to be] distributed through different meetings, instead of doing it all at once. But I also understand that this is sent to patients so that they can do it at home before coming here.” (HCP029, RN in practice for 3 years)

Thus, while *MyDiabetesPlan* promoted a patient-centred approach, the context of the patient’s life and the clinician’s workflow were important factors affecting use of *MyDiabetesPlan*.

#### Theme 1: MyDiabetesPlan appeared to empower patients by providing tailored patient-important information which engaged them in decision-making and self-care. Patients’ use of MyDiabetesPlan was however impacted by their competing medical conditions, other life priorities and socioeconomic context

Patients reported that *MyDiabetesPlan* not only provided patient-relevant education, but also context-specific goals and behavioural support. MyDiabetesPlan “crystallized” for patients the impact of diabetes on their health especially the risk of complications, in a way that prior interactions with clinicians had not:

“What it did for me is to clarify the range of risks that I was dealing with and coming to the conclusion that yes, yes this is the area most that I want to kind of protect against.” (B007, 72-year-old male patient)

*MyDiabetesPlan* allowed for individualized patient-centred goal-setting, and emphasized treating the person not the disease (Table 1):

“It just solidified for me that I was in fact taking the best choice for me personally, not necessarily for my health, but for ME, ME, ME, you know what I mean?” (B007, 72-year-old male patient)

As a result, *MyDiabetesPlan* appeared to engage the patient in her or his care. By bolstering the patient with information and providing a rationale for the action plan, patients reported being more committed and purposeful in their self-care (Table 1):

“That’s what made me, you know, more firm in doing what I was doing. When I started doing it, [it] seems more like common-sensey stuff” (B007, 72-year-old male patient)

Furthermore, patients appeared to be empowered to take an active role in their decision-making with their clinical team (Table 1):

“I found recently that I wasn’t getting proper readings. And so I asked my pharmacist about it. I wouldn’t have done that before, I wouldn’t have questioned her.” (C044, 74-year-old female patient)

Patient education and engagement appeared to facilitate shared decision-making by giving patients a platform to discuss their care with their providers (Table 1):

“I had made notes, I had made goals of what I wanted to change and then I would just check back [on *MyDiabetesPlan*]. So I discussed it with my doctor and the dietician about should we make changes to the medication dosage or type, or should I change the diet back a little bit and moderate that area. So yeah, there was a detailed discussion.” (K017, 50-year-old male patient)

Patients noted that provision of knowledge contributed to balancing out the distribution of responsibility between patients and clinicians:

“It’s [participant’s name]: ‘Pull up your socks and get to work!’ That is what this has taught me. That I can control this. It is up to me.” [B053, 81-year-old female patient]

However, patient engagement with *MyDiabetesPlan* and their diabetes self-management was influenced by competing medical conditions, other life priorities and socioeconomic context.

Patients’ ability to follow *MyDiabetesPlan* and attain their diabetes self-management goals was reportedly impacted by their comorbidities:

“Partly it’s also that I cannot exercise in the same way. I’m a post-polio person. As I’ve aged, I’ve had to bear conditions like arthritis. I’ve also taken several falls that have precluded my being able to walk the way I used to when I didn’t use a cane.” (D066, 74-year-old female patient)

Diabetes also took a back seat also to other life priorities, such as family responsibilities:

“I would try my best to do it, but it’s not always easy. Like now, when my granddaughter has to go to work, I am getting breakfast for the boys, making lunch, sometimes walking them to school, and home again. That sometimes makes it difficult.” (B003, 77-year-old female patient)

Patients’ socioeconomic context factored prominently into accessibility to *MyDiabetesPlan*, including physical access to a computer and internet, health and computer literacy. One patient participant cited diabetes complications and generational issues as reasons for lack of confidence in using a computer on her own:

“I’m not into computers… I know I have everything here [patient holding envelopes with log-in information and handouts]. I keep all the records and that, but I have neuropathy and sometimes I miss things on the computer. […] In my age, we didn’t have the computers that often, so that’s why I don’t feel very comfortable with the computer on my own.” (B053, 81-year-old female patient)

Thus, while *MyDiabetesPlan* appeared to promote shared decision-making, patient engagement in its use was dependent on a myriad of other factors occurring within the appointment and in the context of patients’ lives.

#### Theme 2: MyDiabetesPlan emphasized to clinicians a patient-centred approach that helped patients assume greater ownership for their care. Clinicians’ use of MyDiabetesPlan was impacted by pre-existing clinical tools/workplans, workflow, technical issues, clinic administrative logistics and support, and time. How clinicians adapted to these barriers influenced the degree to which MyDiabetesPlan was integrated into care

*MyDiabetesPlan* enabled clinicians to incorporate new aspects to diabetes care and appeared to reveal novel insights about their patients, particularly around coping with diabetes and support networks, important aspects of a patient-centred approach (Table 1):

“There are some items in *MyDiabetesPlan* that still I haven’t done before in my practice and now I kind of incorporate it in the visit. I actually started applying those things with patients that weren’t part of the study. […] My most favorite part of the tool is as I mentioned in the beginning–the challenge and support. Because sometimes we… ask them but not really in detail. But this one here is written in detail, so I like that.” (HCP029, RN in practice for 3 years)

*MyDiabetesPlan* reemphasized to clinicians the importance of patient-centred education and goal setting:

“It’s all about self-management and patient education and goal setting. And so those are really important pieces. And I think sometimes we tend to go the other way because it’s all about numbers. So this is just a really nice combination that would just meet all of the requirements of good diabetic education.” (HCP037, RN in practice for 39 years)

Similarly to patients, clinicians noted that provision of knowledge helped to balance out the weight of responsibility between patients and clinicians:

“It probably provided my patients with a little bit more information that maybe swung the balance more towards them in terms of responsibility.” (HCP003, FD 9 years in practice)

Further, it highlighted for clinicians the “re-balancing” between clinician beneficence and patient autonomy:

“It in some way strikes the very heart of this continual conflict we have in medical ethics around beneficence versus autonomy. A lot of that will be contingent on capacity: people’s understanding of the disease processes. It’s not to say that when the two are in conflict that autonomy wins out. […] But it probably does remind us that there are still elements of both and that they need to be rebalanced. I think that’s kind of what it did for me.” (HCP038, FD in practice for 8 years)

Thus, clinicians also found that *MyDiabetesPlan* reminded them to reflect on a patient-centred approach to care.

However, just as patients were distracted from using *MyDiabetesPlan* because of competing medical issues, so too were clinicians; diabetes was often not the patient’s priority during the appointment:

“My patients didn’t sustain [use of *MyDiabetesPlan*], and partly that was me because I was so distracted by other, you know, their presentation. They didn’t just come in for their diabetes—they came in for other stuff and they kind of got rolled together and then it was all mixed up.” (HCP025, FD in practice for 37 years)

Use of *MyDiabetesPlan* was sometimes given less priority than pre-existing tools and workflow. Clinicians already had a set way of conducting diabetes care during an appointment:

“I struggle with having a lengthy and structured discussion. It’s never been my interview style. It wasn’t part of my workflow.” (B023, FD in practice for 7 years)

Having prioritized their own algorithm for diabetes care, if they ran out of time, clinicians did not ask their patients to return to clinic in order to complete *MyDiabetesPlan* (Table 1).

Furthermore, lack of integration into their electronic medical record (EMR) negatively impacted their usual workflow:

“I mean, it can’t be a webpage, right? It has to be integrated right into our EMR. ‘Cause as soon as I get taken out of my EMR, that’s just extra time and effort for me, which is problematic, right?” (HCP074, FD in practice for 22 years)

Other technical issues relating to accessibility hampered its’ use by clinicians, particularly in the setting of limited time (Table 1). The process of recalling patients for regularly scheduled diabetes-specific visits, as well as arranging for appropriate appointment time and timing for interprofessional completion of *MyDiabetesPlan* in the clinic was administratively complex and resource-intensive. In some cases, there were no diabetes-specific visits, either by intentional design, or lack of clinic recall systems (Table 1).

Booking an initial appointment with the non-physician team member, to be followed by an appointment with the physician, then informing the patient required significant administrative buy-in and support.

Thus, preexisting clinic workflow, tools, structure and practices often forced out routine implementation of *MyDiabetesPlan* (Table 1).

Clinician participants identified that time was the largest barrier to not only using *MyDiabetesPlan*, but providing diabetes care in general. Time was used up not only in conducting numerous clinical tasks, but also by administrative issues. Although clinicians recognized that care–including goal-setting–takes time, they were powerless to control this finite resource, and worked under its continuous pressure.

Clinicians reported that appointments themselves and the length of the appointment were precious and limited:

“I see my diabetic patients every 3 months at least. Sometimes I have to almost yank them back to see them every 3 months. But there is a lot of work we have to do within that period of time, and usually those are 15-minute appointments.” (HCP003, FD in practice for 9 years)

Lack of time was identified as the largest barrier to completing *MyDiabetesPlan*:

“But time I would say was the biggest factor. Time and resources, so resources being other staff to be able to walk patient through all that information, and time being the amount of time that the visit took.” (HCP003, FD in practice for 9 years)

Clinicians acknowledged that providing diabetes care took time. Not only did actual clinic care take time, but “real life” issues (such as the time to take off shoes) also took up time and impeded timely completion of required tasks (Table 1). They identified that goal-setting requires reflection, which also required time:

“And I think it’s just a matter of actually doing a longer, more detailed conversation with them [patients], and really asking how they feel about the goal that they have. And what happens is as I said, it’s a very long conversation.” (HCP029, RN in practice for 3 years)

To compound this, clinicians struggled with the finiteness of time and the pressured nature of running a clinic:

“If the patient’s even five minutes late, and then you get them in there and then you settle them. And you start talking about going through the tool and the tool takes twenty minutes. Or if they go off on tangent, then you have like five minutes left. And that’s when they tell you about the sore on their foot. Or, the machine isn’t working so you have to fix that or look at it. And then your next appointment, who has taken the time to make the effort to be here on time.” (HCP037, RN in practice for 39 years)

Furthermore, clinicians also identified that it was not only the quantity of time that was important, but that the right “moment” in time was also critical. They postulated that use of *MyDiabetesPlan* could be individualized and leveraged at certain milestones:

“You could do this with certain patients, right? But maybe it’s a couple of appointments of the year, different discussion and you sort of fold it in. […] That’d be interesting. So you’d do it at diagnosis, and then you’d kind of do it at like 2 years, and then at 5 years. Sort of do it progressive across the continuum and see.” (HCPC050, RD in practice for 20 years)

Similarly, particular moments in the patient’s disease and life trajectory may call for use of *MyDiabetesPlan*. One clinician reflected that in retrospect, *MyDiabetesPlan* would have been an effective kickstarter for his/her patient with depression (Table 1).

In light of the finite nature of time quantity, clinicians also pondered over distributing *MyDiabetesPlan* over several appointment, or having patient pre-complete it at home, thus leveraging the “right” time (Table 1). Thus, while the amount of time was important for some, others felt that capturing the “right” moment, in the person’s disease and life trajectory, be it during or outside of the appointment, was critical to meaningful use of *MyDiabetesPlan*.

Clinician participants approached the issue of “time” in different ways. Some clinicians re-framed the purpose of the appointment and *MyDiabetesPlan* such that the time was “justified”:

“It would be the focus of the appointment. It would take up the entire appointment time which, I mean, if the goal of the appointment is to walk through management, goals of care, values, etcetera, then I think that’s fair. But you would need to dedicate a specific appointment to it. It would not be between diabetes follow-up plus this. I think I would have to say: we would have to walk through a *MyDiabetesPlan* today, and this is what we’re dedicating our appointment to.” (HCP074, FD in practice for 22 years)

Similarly, perceived “length” of *MyDiabetesPlan* depended on the clinicians’ perspective regarding “appropriate” appointment lengths:

“I tend to give patients the full fifteen, sometimes twenty minutes for an appointment. Other providers might have found it too long if they are more accustomed to giving 5–7 minutes to a patient.” (HCP074, FD in practice for 22 years)

Thus, clinicians’ “baseline expectations” impacted whether they viewed MyDiabetesPlan as excessively long.

As described above, some clinicians approached the issue of time as an absolute barrier to use, and that using MyDiabetesPlan slowed down the appointment. And yet, “slowing down” can be viewed negatively or positively. Another clinician participant also identified that using MyDiabetesPlan did slow him down:

“But the times when I did [use MyDiabetesPlan], it did slow me down a bit.” (HCP038, FD in practice for 8 years)

However, the participant continued by reflecting on the value of slowing down:

“And arguably in a good way, in that it reminded me that there were a number of assumptions that I was making on behalf of the patients around what their goals might be. It also forced me to be more specific with my language, to be clearer with the language especially when it became overly medicalized.” (HCP038, FD in practice for 8 years)

Thus, given the same context or situation, clinicians appeared to adopt different mindsets. Depending on the mindset adopted, clinicians changed their practices accordingly. For example, one clinician recognized yet embraced that care took time. Her action in response to this was to book longer appointments (Table 1):

“So I think if I were using this tool on a regular basis, I would want to make my appointments an hour. And they’d have to be an hour just to give me that leeway. For some, it was in and out, right? But I didn’t have a lot of young people and so, they just settle in, right? Because they like to be here and they love to chat, right?” (HCP037, RN in practice for 39 years)

Another example of changes in practice that enabled facilitatory actions and behaviours was adapting to the “competition” between pre-existing diabetes tools and *MyDiabetesPlan* described above. While some clinicians interpreted the redundancy of multiple diabetes tools or visit templates as a barrier, others were able to adapt their visit pattern to incorporate the utility of both. This clinician initially had difficulty integrating the two:

“At the beginning, it’s hard because you’re not familiar with the tool. So basically I just do my own thing and then insert the diabetes tool.” (HCP029, RN in practice for 3 years)

However, the clinician continued on to describe how he later adapted to it with greater familiarity:

“But later on, after seeing other patients, I can actually kind of insert it at other parts, like for example, I have my own template right, so after discussing all their bloodwork, their results, I go to their lifestyle, diet and exercise. So from that, I can actually open the diabetes tool ‘cause you have a detailed discussion, detailed questions about diet and exercise. So basically you can actually jump from your own template in the EMR [electronic medical record], and then insert and incorporate part of the diabetes tool, and go back to your EMR, and you know, do the rest, and then go back to the MDP. I think you can really incorporate it too.” (HCP029, RN in practice for 3 years)

Thus, clinicians’ adaptation to perceived barriers, in particular—time, were pivotal to whether and how *MyDiabetesPlan* was integrated into practice.

## Discussion

Together, our interviews with patients and clinicians suggested that *MyDiabetesPlan* appeared to empower patients, engage them in decision-making and help them assume greater ownership for their care. Just as patients’ commitment to their diabetes self-management and *MyDiabetesPlan* was influenced by competing medical conditions, other life priorities and socioeconomic context, clinician engagement with *MyDiabetesPlan* was impacted by pre-existing clinical tools/workplans, workflow, technical issues, clinic logistics and time.

*MyDiabetesPlan* stimulated patient engagement and fruitful discussion and empowered the patient by providing tailored patient-important information, thus rebalancing responsibility and power and promoting shared decision-making. Our finding that *MyDiabetesPlan* empowered patients and allowed clinicians to share responsibility of diabetes care with patients is consistent with what has been reported in the literature; knowledge alone is inadequate to promote shared decision-making but rather a rebalancing of responsibility and power and perceived capacity to influence the decision-making encounter are required [3]. The latter can be developed with the use of pre-consultation interventions such as coaching sessions (including virtual coaching through mobile technologies) [21] and patient activation [22], the use of which are limited due to resource requirements. Given the global proliferation of mobile health applications for diabetes self-management, enabling a mobile version for patient use may be an opportunity to enhance implementation [23]. An optimized decision aid, such as *MyDiabetesPlan*, that patients can independently complete to guide their treatment choices may bridge this gap.

“Time” proved to be a common theme for both patients and clinicians, both of whom were inundated with competing concerns. Specific to clinicians, time pressure is prevalent and a predictor of clinician burnout [24] and has often been cited to be the dominant barrier to SDM [25]. Our study supports such claims but also offer two unique insights: “reframing” clinician’s expectations of time; and the concept of *kairos* (the “right” time). Some clinicians reframed the perceived lack of time: whether or not SDM took “too much time” was relative to the purpose of the encounter (e.g. not too long for goal-setting) or the length of the booking (e.g too long for a 5-minute booking). In addition to this quantitative conceptualization of time (*kronos*), some clinicians considered a qualitative nature of time (*kairos*) that emphasized instead the opportune time to use *MyDiabetesPlan*, relative to the patient’s concurrent issues and disease trajectory. Future scale-up should leverage these insights by emphasizing the goal-setting nature and “timing” of SDM in clinician training materials, as well as targeting administrators and policy makers regarding appointment bookings and remuneration models [26].

Using the concepts of complexity science availed to us two opportunities that we would otherwise have missed. Firstly, sense-making was critical to clinicians’ reception of *MyDiabetesPlan*; whether they perceived it as a useful adjunct, a threat, or redundant tool determined whether they welcomed or rejected it, and what actions they took (or didn’t take) to accommodate its use. Clinician participants exhibited different sense-making, and this subsequently dictated their behaviour (or self-organization) related to *MyDiabetesPlan*. Positive sense-making and adaptive self-organization employed by users can be leveraged in the future to optimize scale-up and sustainability. For example, some clinicians recognized the value of, and time required for, goal-setting and subsequently booked extra time for these appointments; we can optimize integration by leveraging opinion leaders and clinical champions, clinician professional development regarding goal-setting and administrative leadership support to advocate for longer appointment times.

Secondly, identification of actors (patients, clinicians) and what characteristics and factors influenced their behaviours revealed to us that we neglected to interview other key actors that would shed insight into integration of MyDiabetesPlan. For patients, our finding regarding the impact of family responsibilities and computer literacy identified that family members were potential actors that influenced use of MyDiabetesPlan. For clinicians, our finding regarding limited appointment times and complex workflows identified that senior administrative leadership (e.g. medical directors, chronic disease managers), clinic booking staff and information technologist were key actors to reducing technologic barriers and optimizing workflow-related issues. A scoping review confirmed the dearth of studies investigating organizational- and system-level factors but did identify the potential importance of leadership, culture, resources, priorities, teams and workflow characteristics in successful implementation of PtDA [27]. Subsequent work will focus on these factors.

Study limitations include small sample size, particularly of patients. This was due in part to the complexity of our patient population and the challenges with patient recruitment and retention [28] of patients with multiple comorbidities [29]. However, the team believed we had achieved saturation of themes as no new codes nor themes were generated with final interviews in the current sample [30]. Study strengths include our analytic rigour with two independent analysts under the mentorship of an individual with qualitative expertise and application of complexity science as a theoretical perspective for interpreting our data.

In conclusion, *MyDiabetesPlan* appeared to help patients assume greater ownership for their care. Just as patients’ commitment to their diabetes self-management and *MyDiabetesPlan* was influenced by competing priorities, clinician engagement with *MyDiabetesPlan* was impacted by competing clinical priorities and time. Next steps include expanding our interviews to include other key actors (family members, administrative leadership and support, information technology), development or adaptation of clinician professional development resources, as well as formal assessment and design for scaleability and sustainability.

## Supporting information

S1 Table Characteristics of interview participants (clinicians). (DOCX)

S2 Table Characteristics of interview participants (patients). (DOCX)

S1 Appendix Semi-structured interview guide (patient). (DOCX)

S2 Appendix Semi-structured interview guide (health care provider). (DOCX)
