# Supplementary material for: “In my age, we didn’t have the computers”: Using a complexity lens to understand uptake of diabetes eHealth innovations into primary care—A qualitative study
Source: PLoS One. 2021 Jul 7;16(7):e0254157. doi: 10.1371/journal.pone.0254157 (PMC8263251; doi:10.1371/journal.pone.0254157)
Supplement: S1 Table — (DOCX) [file pone.0254157.s001.docx]

**S1 Table. Characteristics of interview participants (clinicians).**

| **Clinician** | **N=10** |
| --- | --- |
| **Sex at birth** | |
| Female | 6 |
| Male | 3 |
| Prefer not to answer | 1 |
| **Duration in Practice (years)** | |
| 2 to 5 | 1 |
| 6 to 10 | 3 |
| Greater than or equal to 16 | 6 |
| **Number of diabetes patients seen per week** | |
| Less than 10 | 5 |
| 10+ | 3 |
| **Type of health team** | |
| Community | 2 |
| Academic | 8 |
