# Supplementary material for: “In my age, we didn’t have the computers”: Using a complexity lens to understand uptake of diabetes eHealth innovations into primary care—A qualitative study
Source: PLoS One. 2021 Jul 7;16(7):e0254157. doi: 10.1371/journal.pone.0254157 (PMC8263251; doi:10.1371/journal.pone.0254157)
Supplement: S2 Table — (DOCX) [file pone.0254157.s002.docx]

**S2 Table. Characteristics of interview participants (clinicians).**

| **Patients** | **N=7** |
| --- | --- |
| **Age** | |
| 45 to 54 years | 1 |
| 65 to 74 years | 4 |
| 75 to 84 years | 2 |
| **Sex at birth** | |
| Female | 4 |
| Male | 3 |
| **Language** | |
| English | 6 |
| Other | 1 |
| **Ethnicity** | |
| White/Caucasian | 5 |
| Other | 2 |
| **Education** | |
| High school or less than high school | 3 |
| Bachelor or post-graduate | 4 |
| **Employment** | |
| Retired | 5 |
| Full time with employee health benefits | 1 |
| Other | 1 |
| **Living arrangements** | |
| Alone | 2 |
| With partner/spouse | 3 |
| With roommates | 1 |
| Other | 1 |
