# Supplementary material for: “In my age, we didn’t have the computers”: Using a complexity lens to understand uptake of diabetes eHealth innovations into primary care—A qualitative study
Source: PLoS One. 2021 Jul 7;16(7):e0254157. doi: 10.1371/journal.pone.0254157 (PMC8263251; doi:10.1371/journal.pone.0254157)
Supplement: S1 Appendix — (DOCX) [file pone.0254157.s003.docx]

**S1 Appendix. Semi-structured interview guide (patient)**

Thank you for taking the time to meet with me today. My name is_____________ and I work with Dr. Catherine Yu from St. Michael’s Hospital (and other docs from Sunnybrook etc). We are conducting interviews with patients with diabetes, and their family members, who participated in our study using our decision aid “My Diabetes Plan”. We are also interviewing the health care providers who used the MyDiabetesPlan. The goal of this research project is to increase the involvement of people with diabetes in decisions about their health care. Findings from these interviews will help us learn how people with diabetes use it. Specifically, we will discuss whether you used the MyDiabetesPlan, how you used the MyDiabetesPlan, why you used it (or not), and what prevented or made it easier for you to use.

The interview should take about 45 to 60 minutes. I will be taping the interview so that I don’t miss any of your comments. All of your comments will remain confidential and will only be accessed by members of the research team. You don’t have to respond to any question that makes you uncomfortable and you may stop the interview at any time.

Have you had a chance to read the consent form? Do you have any questions about the consent or about what I have just explained to you? Are you willing to participate? Great, let’s start.

**[Shared Decision-making and Goal-setting]**

1. In the last 1 year, how have decisions or treatment plans about your diabetes been made in general without use of the MyDiabetesPlan? (e.g. decisions are made together with a doctor or nurse or dietitian, the doctor or nurse or dietitian makes the decision, the doctor or nurse or dietitian offers options and allows you to make the decision)

**Prompts:**

- 1. How important is it for you to participate in making decisions about your diabetes care? Can you tell me more about that?
  2. What did you think about the way these decisions were made? Are there decisions that would benefit most from IPSDM? Or some that IPSDM shouldn’t be used?
  3. How comfortable were you with the way these decisions were made?

How did use of the MyDiabetesPlan affect how decisions were made?

1. In the last 1 year, have you set any goals about your diabetes care? For example, you might have said “I’d really like to stay active and be able to be out and about”. How have you set goals about your diabetes care? (e.g. decisions are made together with a doctor or nurse or dietitian, the doctor or nurse or dietitian makes the decision, the doctor or nurse or dietitian offers options and allows you to make the decision) [review patient’s goals/strategies prior to interview and refer to these]’

**Prompts:**

- 1. How important is it for you to set goals about your diabetes care? Can you tell me more about that?
  2. What did you think about the way these goals were set?
     1. Prompt: Did you feel that the goal you set was truly your own goal?
  3. What made it easier to set goals? What made it harder?
  4. How did use of the MyDiabetesPlan affect how goals were set?

**[MyDiabetesPlan - use]**

1. Do you remember using this website? Does this look familiar? [have website open] If so, what do you remember about it?

If not, go to question 8.

1. Do you remember using this handout? How did you use this? [have patient 1-page handout]
2. Do you remember watching this video? How did you use this? [have patient video open and play start]
3. [If used it] Tell me about how you used or continue to use the “My Diabetes Plan”

**Prompts:**

- 1. Why did you use it? What was your motivation for using the site?
  2. Did you use the My Diabetes Plan l with a health care provider, by yourself, or both?
     1. If you used it with a health care provider, which ones?
        1. How did you use the MyDiabetesPlan with your health care provider?
     2. If you used it by yourself, where did you use it? [at home, waiting room] Did anyone at home help you with it? If so, who?
        1. How did you use the MyDiabetesPlanl on your own? Did you feel comfortable using thewebsite on your own? If not, why not?
  3. How many times did you use it?
     1. [If more than 1 time] Why did you go back to using the site?
        1. How did you use the MyDiabetesPlan when you returned to using it?
     2. [If only one time, or stopped using] If you visited the site only once, what kept you from visiting it again?
     3. What would have encouraged you to visit the site more often? Incentives?

**[MyDiabetesPlan – acceptability of use]**

1. Tell us your overall impressions of this website. (What worked about the website, what didn’t work, and how could it be improved?)

**Prompts:**

- 1. I have the website open on this computer – if you remember, please show me which pages you used on the website and tell me what you liked and disliked about each of these pages.
  2. What content was particularly helpful (or not helpful)? Was the information clear?
  3. How did your previous knowledge about diabetes affect the way you used the MyDiabetesPlan? Ex. Some patients said that the MyDiabetesPlan helped them to request bloodwork from his/her physician. What is your experience?
  4. Do you think the MyDiabetesPlan addressed both the positive and negative aspects of X? Explain.
  5. Did it help you discuss with your health care provider values/concerns that are important to you? How did it do this? How did you feel that your health care provider addressed any values or concerns you raised using the MyDiabetesPlan?
  6. Did you find that MyDiabetesPlan addressed all of the concerns related to diabetes? If not, what would you have liked the MyDiabetesPlan to include?
  7. Did using the MyDiabetesPlanl change the flow of your appointments? If so, how?

**[MyDiabetesPlan - non-use]**

1. [If didn’t remember using it] We are doing this study to find out if people would use it. Why did you not use the MyDiabetesPlan?

**Prompts:**

- 1. Recognizing that all of us have many demands on our attention and time, what do you feel prevented you from using MyDiabetesPlan? (prompts: didn’t know about it? Competing “life” issues? Competing “health” issues? No interest? No time? Not useful? Better sites? Difficult to use? Poor explanations? Not relevant to you? Simply reiterates what the HCP already says?)

**[MyDiabetesPlan - facilitators and barriers to use]**

1. What would have made you use the MyDiabetesPlan more?

**Prompts:**

- 1. How would encouragement from your physician or other health care providers to use the site have affected how often you visited this site?
  2. How would allowing family members or friends access to the site have an impact on your use of the site?
  3. What would make it easier for you to use this website?

1. What made it hard for you to use the MyDiabetesPlan?
2. How did the time it took to complete the MyDiabetesPlan affect your use of MydiabetesPlan?

**[MyDiabetesPlan – Longitudinal Use]**

1. One of the intentions when we designed this website was to create a MyDiabetesPlan that patients could use over time. Based on your comment above you visited the website once/twice…Is there something that we could have done that you would use more often? What would keep you coming back in the future?

**Prompts:**

- 1. What did you do when you received an email from the website about going back to the website to review your diabetes goals?
  2. What would you tell other people with diabetes about this website?
  3. Are you still working towards your goal(s)?
     1. If yes, do you refer back to your goal(s) on the website?

**[Interprofessional approach]**

1. In the last 1 year, who has helped you manage your diabetes?

**Prompts:**

- 1. How important was it for you to have different health care providers help you manage your diabetes? Explain.
  2. How comfortable were you with having different health care providers help you manage your diabetes and set goals?
  3. With use of the MyDiabetesPlan what is the role of the MD? The nurse? The dietician? Other healthcare professionals?

**[Multimorbidity]**

1. You have diabetes and at least two additional chronic conditions. MyDiabetesPlan addressed only one of your conditions, diabetes. As you have these other chronic conditions, how did the websitel help or not help you decide which treatments to start with?

**Prompts:**

- 1. People with diabetes and other chronic disease have many different behavior/lifestyle changes to make. What behavior/lifestyle modifications have you made and how did you decide to make these changes? Did you have to choose one behavior over another depending on one condition being more important than another? Can you give us an example?
