# Supplementary material for: “In my age, we didn’t have the computers”: Using a complexity lens to understand uptake of diabetes eHealth innovations into primary care—A qualitative study
Source: PLoS One. 2021 Jul 7;16(7):e0254157. doi: 10.1371/journal.pone.0254157 (PMC8263251; doi:10.1371/journal.pone.0254157)
Supplement: S2 Appendix — (DOCX) [file pone.0254157.s004.docx]

**S2 Appendix. Semi-structured interview guide (health care provider)**

Thank you for taking the time to meet with me today. My name is_____________ and I work with Dr. Catherine Yu from St. Michael’s Hospital (and other docs from Sunnybrook etc). We are conducting interviews with patients with diabetes, and their family members, who participated in our study using our MyDiabetesPlan “My Diabetes Plan”. We are also interviewing the health care providers who used the MyDiabetesPlan. The goal of this research project is to increase the involvement of patients in decisions about their health care. Findings from these interviews will help us learn how patients use it.

Specifically, we will discuss whether you used the MyDiabetesPlan, how you used the MyDiabetesPlan, why you used it (or not), and what prevented or made it easier for you to use.

The interview should take about 45 to 60 minutes. I will be taping the interview so that I don’t miss any of your comments. All of your comments will remain confidential and will only be accessed by members of the research team. You don’t have to respond to any question that makes you uncomfortable and you may stop the interview at any time.

Have you had a chance to read the consent form? Do you have any questions about the consent or about what I have just explained to you? Are you willing to participate? Great, let’s start.

**[Shared Decision-making and Goal-setting]**

1. In the last 1 year, how have health care decisions or treatment plans about your patient’s diabetes been made? (e.g. decisions are made along with another health care professional? another health care professional makes the decision? you make that decision? You or other health care professionals offer options and allows the patient to make the decision)

**Prompts:**

- 1. How important is it for you that your patient participates in making decisions about his or her diabetes care? What are the pros and cons of this approach for you?
  2. What did you think about the way these decisions were made? ? Are there decisions that would benefit most from IPSDM? Or some that IPSDM shouldn’t be used?
  3. How comfortable were you with the way these decisions were made?
  4. How did use of the MyDiabetesPlan affect how decisions were made?

1. In the last 1 year, how have you helped your patient set goals about his/her diabetes care? (e.g. decisions are made along with another health care professional? another health care professional makes the decision? you make that decision? You or other health care professionals offer options and allows the patient to make the decision)

**Prompts:**

- 1. How important is it for you to help your patients to set goals about his/her diabetes care?
  2. What did you think about the way these goals were set?
  3. What made it easier to set goals? What made it harder?
  4. How did use of the MyDiabetesPlan affect how goals are set?

**[MyDiabetesPlan - use]**

1. Do you remember using this decision-making aid? Does this look familiar? [have decision making aid open] If so, what do you remember about it?

If not, go to question 8.

1. Do you remember using this? How did you use this? [have provider 1-page handout]
2. Do you remember watching this? How did you use this? [have provider video open and play start]
3. [If used it] How did you use it?

**Prompts:**

- 1. Why did you use it? What was your motivation for using the site?
  2. How many times did you use it?
     1. [If more than 1 time] What kept you returning to the site? Tell me about that. Why did you go back to using it? How did you use the MyDiabetesPlan when you returned to it?
     2. [if only one time, or stopped using] If you visited the site only once, what kept you from visiting it again?
     3. What would have made you visit the site more often? Incentives?

**[MyDiabetesPlan – acceptability of use]**

1. Tell us your overall impression about the website (What worked about the website, what didn’t work, and how could it be improved?)

**Prompts:**

- 1. I have the decision-making aid open on this computer – please show me which pages you used on the decision-making aid and tell me what you liked and disliked about each of these pages.
  2. Do you think the MyDiabetesPlan provided a balanced or unbalanced view of risks and benefits, pros and cons? If not, please explain why.
  3. Did it help your patient express values that were important to him or her? How did it do this? Can you give us an example of when this occurred?
     1. How did you address the values/concerns the patient brought up while using the MyDiabetesPlan? Can you give an example of when you responded to the patient’s concerns?
  4. Did it help you to learn more about your patient? Can you give an example?
     1. Some of our users noted that it could be used as a motivational MyDiabetesPlan for patients as it allowed you to focus on problem areas. What is your experience?
  5. Did it help you talk through your patient’s diabetes care? How did it do this?
     1. For example, did the MyDiabetesPlan help you to discuss treatment options or the patient’s health status?
     2. Some people reported that it was an opportunity to provide patient education. What was your experience with this?
     3. Some people reported that it was an opportunity to provide the patient with supports. What was your experience with this?
     4. Some people reported that it was an opportunity to set management plans with the patient. What was your experience with this?
  6. Can you comment on the ease of use of the MyDiabetesPlan? (e.g. how clear or not clear was it?)
  7. How did you find the length of time it took to complete the MyDiabetesPlan? Too long? Too short? Just right?
  8. How did use of the MyDiabetesPlan affect the flow of your appointments?
     1. How did you integrate use of the MyDiabetesPlan into your appointments? (Prompt: Did you use it as a prompt? A cue? A template? A conversation aid? As a behavior change/ engagement MyDiabetesPlan?) What helped/hindered you in integrating the MyDiabetesPlan?
     2. How did the MyDiabetesPlan help you to gather your history from your patient?
     3. How did you bring up use of the MyDiabetesPlan with your patients?
     4. At what point in an appointment would you use the MyDiabetesPlan?
     5. How did you apply the plan in future appointments?
     6. How did the MyDiabetesPlan affect patient rapport? How did this affect your ability to conduct shared decision making? Goal setting?

**[MyDiabetesPlan - non-use]**

1. [If didn’t remember using it] We are doing this study to find out if people would use it. Why did you not use the MyDiabetesPlan?

**Prompts:**

- 1. Recognizing that all of us have many demands on our attention and time, what do you feel prevented you from accessing the site? (prompts: No interest? No time? Not useful? Better sites? Poor explanation? Redundancy? Difficult to use? Tough questions to ask patient? Not applicable to your patients?)

**[MyDiabetesPlan - facilitators and barriers to use]**

1. What would have made you use the MyDiabetesPlan more?

**Prompts:**

- 1. How would encouragement from your patient, patient’s family members, or other health care providers to use the site have affected your use of the site?

1. Did any of your patients use the MyDiabetesPlan on their own?

**Prompts:**

- 1. If yes, how did this affect the use of the MyDiabetesPlan in the appointment?
  2. In your experience, were patients able to correctly answer questions when using the MyDiabetesPlan on their own?

1. What would make it easier for you to use this MyDiabetesPlan?
2. What made it hard for you to use this MyDiabetesPlan?

**[MyDiabetesPlan – Longitudinal Use]**

1. One of the intentions when we designed this website was to create a MyDiabetesPlan that patients could use overtime. Based on your comment above you visited the website once/twice… Is there something that we could have done to create a personal webpage that you would use more often?

**Prompts:**

- 1. What would you tell other patients with diabetes about this decision-making aid?
  2. What patients would you recommend use this MyDiabetesPlan in the future? What patients would you not recommend use the MyDiabetesPlan? What patients would you recommend participate in IPSDM? Are there any patients you would suggest not participate in IPSDM?

**[Interprofessional approach]**

1. In the last 1 year, who has helped your patient manage his/her diabetes?

**Prompts:**

- 1. How important was it to have other health care providers help manage his/her diabetes?
  2. What did you think about this approach to helping your patient manage his/her diabetes?
  3. How comfortable were you with having different health care providers help your patient manage his/her diabetes?
  4. With use of the MyDiabetesPlan what is the role of the MD? The nurse? The dietician? Other healthcare professionals?
  5. Is there any specific setting (i.e. practice type) or appointment type you think the MyDiabetesPlan would be most useful?

**[Multimorbidity]**

1. Many of your patients have diabetes and at least two additional chronic conditions. This MyDiabetesPlan addressed diabetes and was designed to make diabetes management more focused. Did it help or not help you determine which treatments to start with, given that many of your patients had other chronic conditions? How so?
